# PKC**δ** Regulates Translation Initiation through PKR and eIF2**α** in Response to Retinoic Acid in Acute Myeloid Leukemia Cells

**DOI:** 10.1155/2012/482905

**Published:** 2012-07-15

**Authors:** Bulent Ozpolat, Ugur Akar, Ibrahim Tekedereli, S. Neslihan Alpay, Magaly Barria, Baki Gezgen, Nianxiang Zhang, Kevin Coombes, Steve Kornblau, Gabriel Lopez-Berestein

**Affiliations:** ^1^Department of Experimental Therapeutics, Unit 422, The University of Texas MD Anderson Cancer Center, 1515 Holcombe Boulevard, Houston, TX 77030, USA; ^2^Department of Leukemia, The University of Texas MD Anderson Cancer Center, 1515 Holcombe Boulevard, Houston, TX 77030, USA; ^3^Department of Bioinformatics and Computational Biology, The University of Texas MD Anderson Cancer Center, 1515 Holcombe Boulevard, Houston, TX 77030, USA

## Abstract

Translation initiation and activity of eukaryotic initiation factor-alpha (eIF2**α**), the rate-limiting step of translation initiation, is often overactivated in malignant cells. Here, we investigated the regulation and role of eIF2**α** in acute promyelocytic (APL) and acute myeloid leukemia (AML) cells in response to all-*trans* retinoic acid (ATRA) and arsenic trioxide (ATO), the front-line therapies in APL. ATRA and ATO induce Ser-51 phosphorylation (inactivation) of eIF2**α**, through the induction of protein kinase C delta (PKC**δ**) and PKR, but not other eIF2**α** kinases, such as GCN2 and PERK in APL (NB4) and AML cells (HL60, U937, and THP-1). Inhibition of eIF2**α** reduced the expression of cellular proteins that are involved in apoptosis (DAP5/p97), cell cycle (p21Waf1/Cip1), differentiation (TG2) and induced those regulating proliferation (c-myc) and survival (p70S6K). PI3K/Akt/mTOR pathway is involved in regulation of eIF2**α** through PKC**δ**/PKR axis. PKC**δ** and p-eIF2**α** protein expression levels revealed a significant association between the reduced levels of PKC**δ** (*P* = 0.0378) and peIF2 (*P* = 0.0041) and relapses in AML patients (*n* = 47). In conclusion, our study provides the first evidence that PKC**δ** regulates/inhibits eIF2**α** through induction of PKR in AML cells and reveals a novel signaling mechanism regulating translation initiation.

## 1. Introduction

Differentiation block or arrest is one of the major characteristics of acute myeloid leukemia (AML) [1]. All-*trans* retinoic acid (ATRA), an active metabolite of vitamin A, is a potent inducer of cellular differentiation and growth arrest in various tumor cell lines and has been successfully used in the treatment of acute promyelocytic leukemia (APL) [1–5]. The success of ATRA in the treatment of APL introduced the concept of differentiation therapy in treating malignant diseases [1]. Arsenic trioxide (ATO), an FDA approved drug, induces both differentiation and apoptosis in APL and AML cells [5]. The molecular events that are involved in underlying mechanism of these drugs are not completely elucidated. Understanding the pathways regulating cell proliferation and differentiation may help designing new molecularly targeted therapies in AML.

Translation initiation is a highly regulated process of translation in response to cellular stress and mitogenic stimulation [6–11]. Increased translation and protein synthesis are associated with cell proliferation and malignant disease [6, 7]. Translational regulation plays a vital role in the expression of oncogenic, and growth-regulatory, differentiation, and apoptosis related proteins and is considered one of the important but understudied feature of malignant phenotype [6–10, 12, 13].

 Increased activity of eukaryotic translation initiation factor-2*α* (eIF2*α*) is the rate-limiting step of translation initiation and phosphorylation of eIF2*α* at serine 51 converts eIF2 to a competitive inhibitor of eIF2B, resulting in the inhibition of translation [6, 13–16]. Transfection of cells with eIF2*α* has been shown to cause malignant transformation of normal cells, suggesting that eIF2*α* plays a critical role in cellular pathways controlling cell proliferation [10, 11, 17–27]. Phosphorylation of eIF2*α* on serine 51 (Ser51) by eIF2*α* kinases, such as PKR, GCN2, and PERK, leads to the increased affinity of eIF2*α* for eIF2B and converts the phosphorylated eIF2*α* into an inhibitor of the GDP-GTP exchange factor, thereby inhibiting eIF2*α* activity and translation initiation [14]. While reducing global translation, phosphorylation of eIF2*α* also induces preferential translation of specific mRNAs that assist in the regulation of genes involved in metabolism and apoptosis [25].

We and others reported that ATRA and ATO inhibit translation initiation through multiple posttranscriptional mechanisms, including downregulation of translation factors and upregulation of repressors of translation initiation, such as PDCD4 and DAP5/p97 in APL cells [28, 29]. However, the posttranscriptional mechanisms regulating in APL and AML cells remain largely unknown.

Protein kinase C (PKC) is a family of serine/threonine protein kinases that are key regulatory enzymes in signal transduction [30]. The PKC family is divided in three groups, based on the differences in their sequence homology and cofactors required for their activation. The conventional PKCs (*α*, *β*I, *β*II, and *γ*) are activated by calcium and 1,2-diacyl-*sn*-glycerol (DAG), whereas the novel class of PKCs (*δ*, *ε*, *θ*, *η*) are calcium independent but DAG dependent. The atypical PKCs (*λ*, *ζ*, and *υ*) do not require calcium or DAG for their activation. Depending on the cell type, PKC*δ* can function as a tumor suppressor, a proapoptotic factor, and can regulate cell proliferation and cell survival functions [30]. The role of PKC*δ* in regulation of translational machinery is not well understood.

In the present study, we investigated the regulation of eIF2*α* in APL and AML cells. We found that PKC*δ* regulates eIF2*α* activity by phosphorylating it at Ser-51 through PKR, an eIF2*α* kinase. We also found PI3K/Akt/mTOR pathway is involved in regulation of eIF2*α* through PKC*δ*/PKR. Overall, our data provided the first evidence that PKC*δ* regulates phosphorylation/activity of eIF2*α* through PKR in APL and AML cells, revealing a novel role of PKC*δ* signaling and regulation of translation initiation.

## 2. Materials and Methods

### 2.1. Cell Lines and Culture Conditions

The human promyelocytic cell line NB4, (AML-M3 type by the FAB classification) harboring *t*(15;17), was purchased from German Collection of Microorganisms and Cell Cultures (Braunschweig, Germany). AML cell lines including myeloblastotic HL60 (M2-AML), myelomonocytic U937 (M4/M5-AML), and THP-1 (M4-AML) cells were purchased from American Type Culture Collection (Manassas, VA). Primary human hematopoietic progenitor cells (CD34+ bone marrow progenitor cells) were purchased from Cambrex Bioscience Inc. (Walkersville, CA). The cells were grown in RPMI 1640 medium (Life Technologies, Carlsbad, CA) supplemented with 10% heat-inactivated fetal bovine serum at 37°C under 5% CO_2_ in a humidified incubator. ATRA, arsenic trioxide (ATO), and rottlerin were purchased from Calbiochem, La Jolla, CA.

### 2.2. Evaluation of Cell Differentiation

Cell differentiation was identified by examining expression of granulocytic (CD11b and CD11c) and monocytic (CD14) differentiation markers, morphologic changes, electron microscopy images, and reformation of PML nuclear bodies. Cells were collected from 2 to 5 days after treatment and washed with phosphate-buffered saline solution (PBS). Cells (5 × 10^5^) in 100 *μ*L of PBS were incubated for 30 min with fluorescein isothiocyanate (FITC)—labeled or phycoerythrin (PE)—labeled anti-CD11b, anti-CD11c, or anti-CD14 antibodies (1 : 200; BD Biosciences, San Jose) and for 20 min with PE-labeled isotope control IgG2a (Becton Dickinson) on ice in the dark, as described by the manufacturer. The percentages of CD11b^+^, CD11c^+^, and CD14+ cells were determined by fluorescence-activated cell sorting (FACS) analysis at the Flow Cytometry and Cellular Imaging Facility, The University of Texas MD Anderson Cancer Center. Background staining was determined from the cells stained with the isotype control antibodies.

### 2.3. Cell Growth Assays

Cells were seeded at 1 × 10^5^ cells/mL in RPMI medium in six-well tissue culture plates (Costar, Cambridge, MA). After dilution with saline from a 10 mM stock in DMSO, cells were treated with ATRA at a final concentration of 1 *μ*M and incubated for 3 days. The maximum concentration of DMSO was kept at less than 0.001% (v/v). Cell viability was determined by two methods: trypan blue (Sigma) exclusion and the 3-(4,5-dimethylthiazol-2-yl)-2,5-diphenyltetrazolium bromide (MTT; Sigma) dye reduction assay [31]. Briefly, after cells were incubated with ATRA or ATO, 10 *μ*L of MTT solution (10 mg/mL in PBS) was added to each well (in triplicate). The plates were then incubated for 4 h at 37°C, and the formazan crystals formed were dissolved by adding 100 *μ*L of 0.04 N HCl in 2-propanol. Plates were read at 490 nm by a microplate reader (Molecular Devices, Sunnyvale, CA). In the trypan blue exclusion test, cells with intact membranes exclude the dye, whereas cells without intact membranes take up the coloring agent. For this assay, a cell suspension was prepared and mixed with an equal amount of a 0.4% trypan blue solution. Cell viability was assessed within 1-2 min by calculating the percentage of unstained (i.e., viable) cells. Untreated controls were used to determine the relative viability in both assays.

### 2.4. Western Blot Analysis

NB4, HL60, THP-1, and U937 cells were harvested from exponentially growing cell cultures. After treatment, the cells were collected, centrifuged, and lysed with a lysis buffer. Total protein concentration of the resulting whole-cell lysates was determined using a DC protein assay kit (Bio-Rad, Hercules, CA). In the experiments in which the PKC*δ* inhibitor rottlerin (4 *μ*M) were used, the cells were incubated with the inhibitor for 4 h and then with ATRA for the indicated time periods. Aliquots containing 30 *μ*g of total protein from each sample were subjected to sodium dodecyl sulfate polyacrylamide gel electrophoresis (SDS-PAGE) and electrotransferred to the membranes as described previously [32]. The membranes were blocked with 5% dry milk in TBST (100 mM Tris-HCL [pH 8.0], 150 mM NaCl, and 0.05% Tween-20), probed with primary antibodies diluted in TBST containing 5% dry milk, and incubated at 4°C overnight. We used primary antibodies against eIF2, phosphorylated (p) eIF2e (Ser51), p-eIF4E (Ser209), p-4E-BP1 (Thr37/40), Akt (Ser473), P-PERP (The981), p-P70S6K (Thr421/Ser424) (all, Cell Signaling Technology, Danvers, MA), and p-PKR (Thr446) (Santa Cruz Biotechnology, Santa Cruz, CA); p21^Waf1/Cip1^, tissue transglutaminase 2 (TG2), and GCN2 also were purchased from Santa Cruz Biotechnology. eIF2*α* antibodies were diluted 1 : 1,000 in TBST. After being washed, the membranes were incubated with horseradish peroxidase-conjugated anti-rabbit secondary antibody (Amersham Life Science, Cleveland, OH). Mouse anti-*β*-actin and donkey anti-mouse secondary antibodies were purchased from Sigma to examine *β*-actin expression for equal loading. The bands were visualized by the enhanced chemiluminescence method (KPL, Gaithersburg, MD). Images were scanned and quantitated by a densitometer using the Alpha Imager application program (both from Alpha Innotech, San Leandro, CA). All experiments including treatments were repeated at least three times.

### 2.5. RNA Isolation and RT-PCR Analysis

Cells were seeded in six-well plates (1 × 10^6^ cells/mL) and treated with ATRA at a final concentration of 1 *μ*M. The cells were collected at various time points, and total cellular RNA was isolated with Trizol reagent (Life Technologies). cDNA was obtained from 5 *μ*g of total RNA using a Superscript II RT kit (Life Technologies) as previously described [32]. Briefly, 5 *μ*L of the total 20 *μ*L of reverse-transcribed product was used for polymerase chain reaction (PCR) in 1× PCR buffer containing 1.5 mM MgCl_2_, 250 *μ*M dNTPs, 0.5 units of Taq polymerase (Life Technologies), and 100 ng of primers for PKC*δ* (Santa Cruz Biotechnology) or *β*-actin (Sigma-Genosys, Houston, TX). The following programs were used for PCR amplification of PDCD4: 1 cycle at 94°C for 2 min; 35 cycles of denaturation at 94°C for 1 min; annealing at 55–65°C for 1 min; extension at 72°C for 1 min. A cycle of 72°C for 7 min was added to complete the reaction. The reaction products were analyzed on 2% agarose gels containing ethidium bromide, and cDNA synthesis was verified by detection of the *β*-actin transcript.

### 2.6. Knockdown of PKC*δ* and eIF2*α* by siRNA

Targeted downregulation of eIF2*α* and PKC*δ* was achieved by using double-stranded small-interfering RNA (siRNA), which were purchased from Santa Cruz Biotechnology and Invitrogen Inc. The control and FITC-labeled siRNA were purchased from Qiagen (Valencia, CA). Transfection of siRNA was performed by using an optimized nucleofection protocol according to the manufacturer's instructions (Amaxa Inc., Gaithersburg, MD). Exponentially growing NB4 cells were harvested and 2 × 10^6^ cells were used for siRNA transfection experiments. Cells were also transfected with control (nonsilencing) siRNA [35]. Under these conditions, we consistently reached a transfection efficiency of 70% without significant reduction of viability in the cell lines. Untransfected cells and cells treated with transfection reagent or control siRNA were used as controls. Target protein expression was determined 24, 48, 72, and 96 h after transfection by Western blot analysis. Fresh medium containing 1 *μ*M ATRA was added for the analysis at 72 h. After the treatment, the cells were harvested to assess differentiation markers by FACS analysis.

### 2.7. Reverse Phase Protein Array (RPPA)

The assay was performed at MD Anderson Cancer Center in collaboration with Dr. Steven Kornblau using AML samples under an approval protocol and consent. Paired samples from the same patients isolated at the time of diagnosis and relapse from the same patients were analyzed by RPPA assay as previously described [36, 37].

#### 2.7.1. Statistical Analysis

The results are expressed as means ± standard deviations of three or more experiments. Statistical analysis was performed using the two-tailed Student's *t*-test for paired data. *P* values less than 0.05 were considered significant. Comparison of the protein levels detected by reverse phase protein array (RPPA) in pairs (each pair are from the same patient) of “newly diagnosed” and “relapsed” AML Samples was analyzed by paired *t*-test to assess the difference.

## 3. Results

### 3.1. Evaluation of ATRA- and ATO-Induced Effects

**Figure 1:**
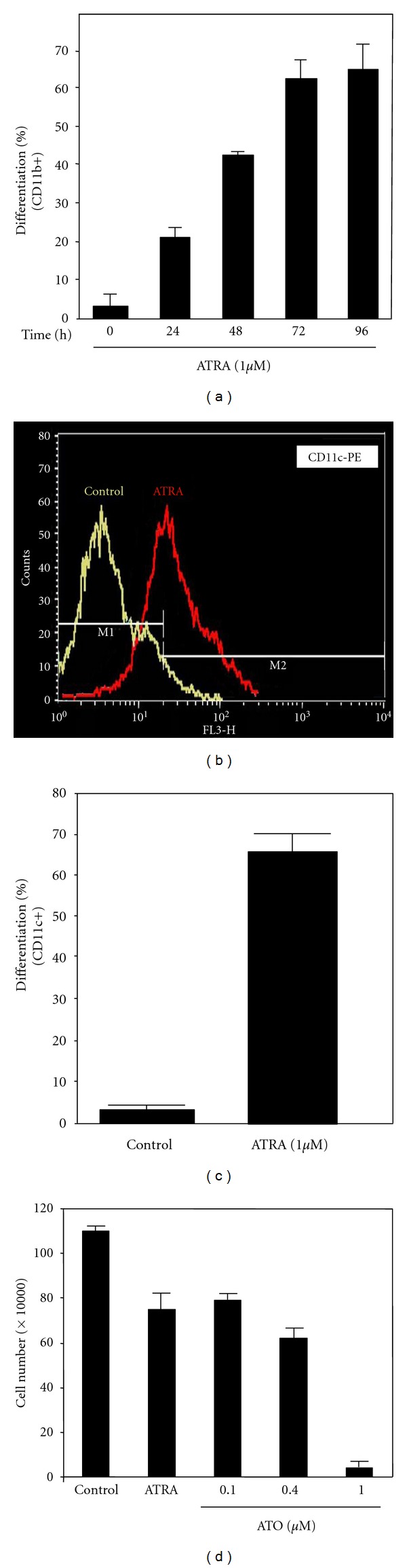
ATRA- and ATO-mediated effects in APL cells. APL (NB4) cells were kept in growth medium with ATRA (1 *μ*M) or without ATRA for the indicated time periods. The cells were stained with monoclonal anti-CD11b (a) or anti-CD11c ((b) and (c)) antibodies to detect induction of granulocytic differentiation and analyzed by flow cytometry. (d) ATO inhibits growth of NB4 cells. NB4 cells were treated with ATO at the indicated concentrations for 48 h and viable cells were counted as described in Section 2. ATO-induced apoptosis was determined by the activation of caspases 9 and 3 as well as cleavage of PARP in NB4 cells (see Supplementary Figure 2 in Supplementary Material available online at doi: 10.1155/2012/482905).

 We first determined the effects of ATTA and ATO on cell differentiation and apoptosis of NB4 cells. NB4 cells (M3-AML) are true APL cells that express *t*(15 : 17) PML-RAR*α*. NB4 cells that were treated with ATRA (1 *μ*M) at the indicated time points underwent granulocytic differentiation, as indicated by induction of CD11b and CD11c expression detected by FACS analysis (Figures 1(a), 1(b), and 1(c)). Induction of differentiation was also evidenced by PML nuclear body reformation (data not shown), May-Grünwald-Giemsa staining and transmission electron microscopy that ATRA-treated cells acquired granulocytic morphology: decreased nuclear/cytoplasm ratio, the appearance of cytoplasmic granules, and nuclear lobulation (Supplementary Figure 1). ATO induced growth inhibition by cell toxicity assay (Figure 1(d)) and apoptosis by activation of caspases 9 and 3 as well as cleavage of PARP was detected (Supplementary Figure 2) [5, 38].

### 3.2. ATRA and ATO Induce Ser-51 Phosphorylation of eIF2*α* in APL Cells

**Figure 2:**
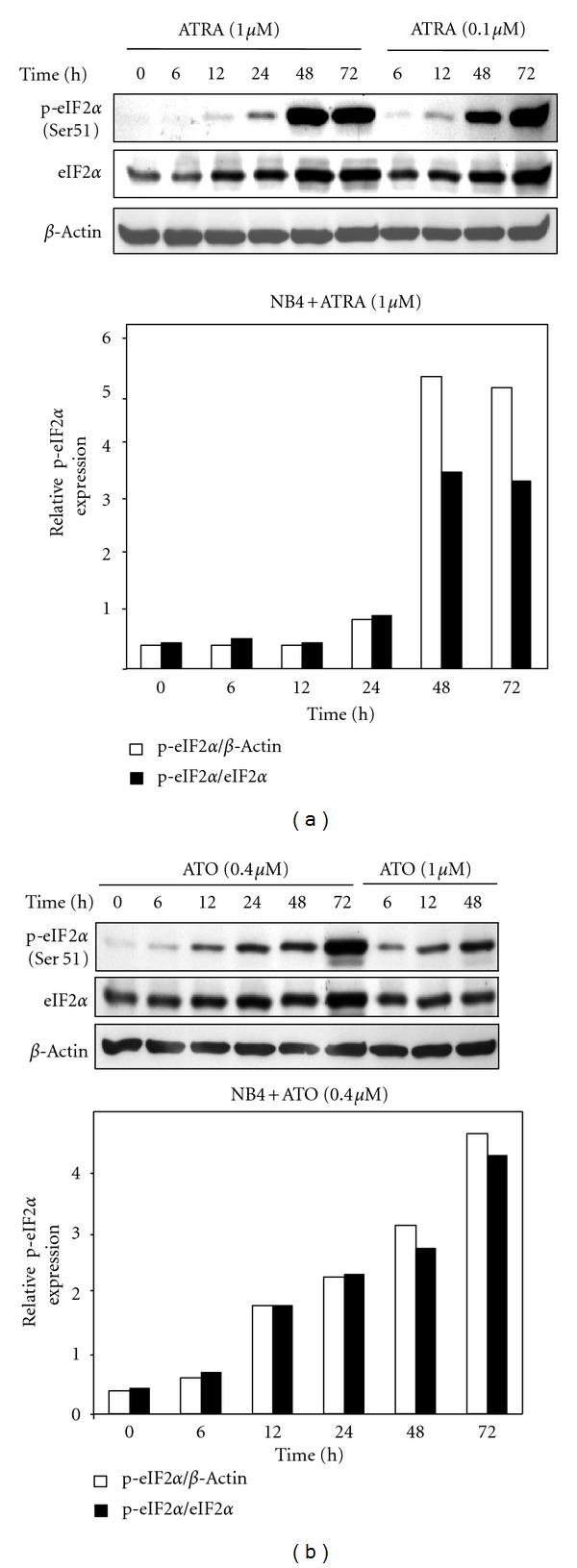
ATRA and ATO induce phosphorylation of eIF2*α*. (a) NB4 cells were treated with ATRA (0.1 *μ*M or 1 *μ*M) for the indicated time periods or left untreated. Equal amounts of total cell lysate were analyzed by SDS-PAGE and immunoblotted with antiphospho (Ser51) eIF2*α* or eIF2*α* antibodies. *β*-Actin was used as loading control for the Western blots. The panel (below) represents densitometric analysis shows relative expression of p-eIF2*α* after normalization to total eIF2*α* and actin levels. (b) ATO (0.4 *μ*M or 1 *μ*M) induces phosphorylation (Ser51) of eIF2*α* and total eIF2*α* in a time-dependent manner in NB4 cells. Densitometry analysis (below) represents relative p(Ser51)-eIF2*α* expression seen in the blots.

Previous studies suggest that growth inhibition and terminal differentiation are associated with inhibition of total protein synthesis and translational suppression by differentiation-inducing agents in APL, AML, and other cells [28, 33, 34, 39, 40]. We have previously reported the evidence that ATRA modulates the expression of proteins involved in suppression of translation initiation during granulocytic differentiation of APL cells [32–34]. Phosphorylation of serine 51 on the alpha subunit of eukaryotic initiation factor eIF2 is a well-documented mechanism of inhibition of translation and global inhibition of protein synthesis under a variety of conditions in variety of cells including APL and AML cells [14–16, 40, 41]. Therefore, we first investigated whether ATRA induces phosphorylation of eIF2*α* for inhibition of translation and protein expression in APL cells. ATRA treatment induced marked phosphorylation (Ser 51) of eIF2*α* in the cells at the differentiation-inducing concentrations of ATRA (0.1 or 1 *μ*M) in NB4 cells (Figure 2(a)). ATRA-induced phosphorylation of eIF2*α* started at 24 h, reached its maximum at 48 h to 72 h of treatment, and correlated well with the level of differentiation observed in NB4 cells (Figure 1(a)). Total and unphosphorylated eIF2*α* expression were also elevated during differentiation. Densitometric analysis showed that p-eIF2*α*/eIF2*α* and p-eIF2*α*/*β*-actin ratios were increased about 7- to 10-fold (Figure 2(a), lower panel). Exposure of NB4 cells to ATO (0.4 and 1 *μ*M) for 6–72 h also resulted in Ser-51 phosphorylation of eIF2*α* in NB4 cells as indicated by Western blot analysis (Figures 2(b) and 2(c)), suggesting that ATRA and ATO have similar effects in inducing phosphorylation of eIF2*α*.

### 3.3. ATRA- and ATO-Induced Phosphorylation of eIF2*α* Is Mediated PKR but Not PERK and GCN2

**Figure 3:**
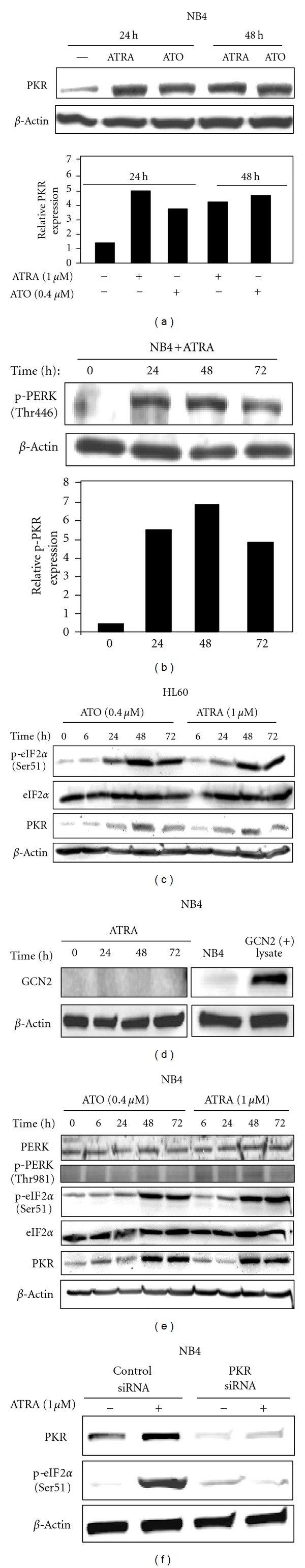
PKR regulates phosphorylation (Ser51) of eIF2*α*. (a) NB4 cells were treated with either ATRA (1 *μ*M) or ATO (0.4 *μ*M) at the indicated time points and PKR levels were detected by Western blot using PKR specific antibody. Densitometry analysis (lower panel) represents relative PKR expression after normalizing to actin expression. (b) NB4 cells were treated with either ATRA (1 *μ*M) at the indicated time points and p-(Thr446) PKR levels were detected by Western blot. (c) HL60 cells were treated with either ATO (0.4 *μ*M) or ATRA (1 *μ*M) at the indicated time points and PERK, p-(Thr981)PERK, p-eIF2*α*, and PKR levels were detected by Western blot. (d) Expression of GCN2, an eIF2*α* kinases, is not induced by ATRA in NB4 cells. GCN2 positive cell lysate were used as positive controls for anti-GCN2 antibody and Western blot analysis. (e) ATO and ATRA do not induce PERK eIF2*α* kinase. NB4 cells were treated with either ATO (0.4 *μ*M) or ATRA (1 *μ*M) at the indicated time points and PERK, p-(Thr981)PERK, p-eIF2*α*, and PKR levels were detected by Western blot. (f) Knockdown of PKR by a specific siRNA blocks the ATRA-induced phosphorylation of eIF2*α* in NB4 cells by Western blot. Bar graph represents inhibition and the relative expression of PKR (PKR/*β*-actin) and p-eIF2*α* (p-eIF2*α*/*β*-actin) after siRNA treatments by densitometry analysis of Western blot bands.

PKR is an eIF2*α* kinase that is known to phosphorylates eIF2*α* on the Ser51 residue [25, 42–44]. To determine whether ATRA- and ATO-induced phosphorylation of eIF2*α* is mediated by PKR activity in APL cells we examined PKR expression. We found that ATRA and ATO induce marked PKR expression in NB4 cells (Figure 3(a)). We also observed activity of PKR as indicated by p-PKR (Thr448) was also induced (Figure 3(b)). We also observed similar effects in HL60 cells that undergo granulocytic differentiation by ATRA and ATO (Figure 3(c)).

GCN2 and PERK induce eIF2*α* phosphorylation in response to stress and unfolded protein response (UPR) [25]. We next examined whether other eIF2*α* kinases, including GCN2 and PERK, are involved eIF2*α* regulation during ATRA. Neither ATRA nor ATO treatment resulted in induction of GCN2 and PERK in NB4 cells (Figures 3(d) and 3(e)). We did not detect any change in PERK levels, suggesting no UPR type of response is involved in the process (Figure 3(e)).

To show a link between PKR and phosphorylation of eIF2*α*, we knocked down PKR by a specific siRNA and found that inhibition of PKR completely blocked ATRA-induced phosphorylation (Ser51) of eIF2*α* in NB4 cells (Figure 3(f)). To further eliminate possibility that PERK is not involved in ATRA-induced regulation of eIF2*α*, we knocked down of PERK by a specific siRNA and did not detect any change in phosphorylation status of eIF2*α* (Supplementary Figure 3). Overall, these findings suggested that PKR plays a major role in the phosphorylation of eIF2*α*.

### 3.4. ATRA and ATO Induce p-Ser51 eIF2*α* and PKR in AML Cells

**Figure 4:**
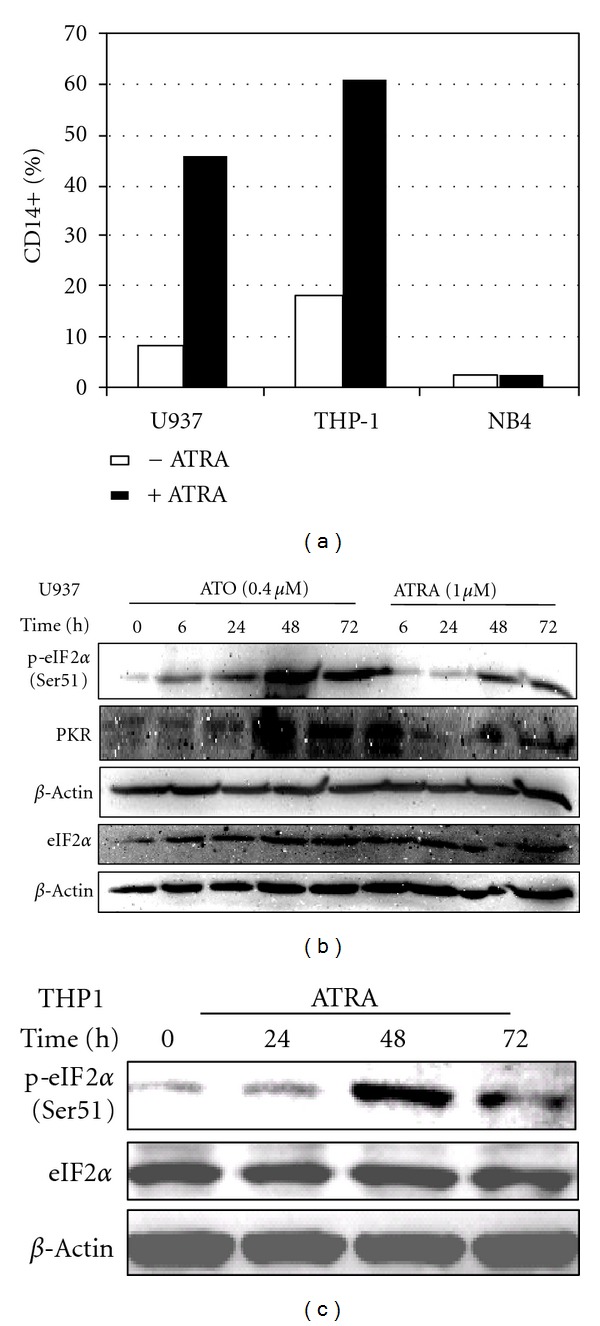
ATRA and ATO induce p-eIF2*α* during monocytic differentiation of AML cells. (a) ATRA (1 *μ*M) induces monocytic differentiation in U937 and THP-1 cells but not in NB4 cells as indicated by induction of CD14 expression on the cells detected by FACS analysis. (b) ATRA and ATO induce phosphorylation (Ser51) of eIF2*α* expression in U937 AML cells. The U937 cells were treated with either ATRA (1 *μ*M) or ATO (0.4 *μ*M) for the indicated time periods and analyzed by Western blot. (c) ATRA (1 *μ*M) also induces phosphorylation (Ser51) of eIF2*α* expression in THP-1 monocytic AML.

We next investigated whether ATRA induces phosphorylation of eIF2*α* in AML cells during terminal differentiation. It is well established that ATRA induces monocytic differentiation of U937 (M4/M5-AML) [45] and THP-1 (M4-AML) [46, 47] cells. Monocytic differentiation of ATRA (1 *μ*M) treatment of U937 and THP-1 cells was shown by CD14 expression by FACS analysis (Figure 4(a)). ATRA (1 *μ*M) treatment of THP-1 and U937 cells led to an induction of phosphorylation (Ser51) of eIF2*α*, which is associated with marked PKR expression (Figures 4(b) and 4(c)). ATO (0.4 uM) also induced p-Ser51 eIF2*α* and PKR expression. These findings provided further evidence that phosphorylation of eIF2*α* is regulated by PKR by ATRA and ATO.

### 3.5. PKC*δ* Is Involved in Ser51-Phosphorylation of eIF2*α* in APL Cells

**Figure 5:**
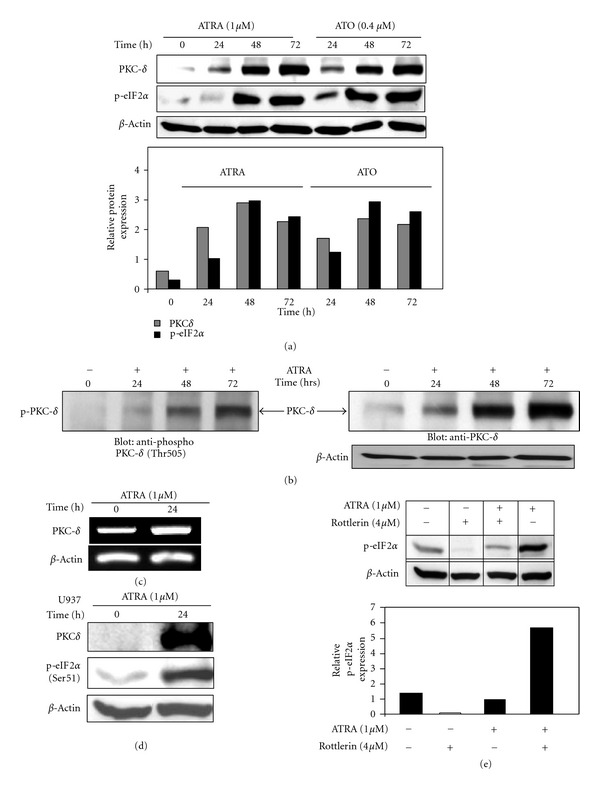
PKC*δ* regulates the phosphorylation (Ser51) of eIF2*α* by ATRA and ATO in APL cells. (a) NB4 cells were left untreated or incubated with ATRA (1 *μ*M) or ATO (0.4 *μ*M), for the indicated time periods. Equal amounts of total cell lysate were analyzed by SDS-PAGE and immunoblotted with specific antibodies against PKC*δ* or p-eIF2*α*. *β*-Actin was used as a loading control. (b) ATRA (1 *μ*M) induces phosphorylation (Thr505) of PKC*δ* at the activation domain detected by Western blot in NB4 cells. (c) ATRA (1 *μ*M) induces PKC*δ* mRNA expression. After 24 h of ATRA treatment, NB4 cells were collected and total cellular RNA was extracted to detect PKC by RT-PCR using specific primers. The reaction products were analyzed on 2% agarose gels. cDNA synthesis and equal loading were verified by detection of the *β*-actin transcript. (d) ATRA (1 *μ*M) induces phosphorylation of eIF2*α* in U937 (M4/M5-AML) cells. (e) Inhibition of PKC*δ* by rottlerin inhibits basal and ATRA-induced phosphorylation (Ser51) of eIF2*α*. Exponentially growing NB4 cells were collected and pretreated with specific PKC*δ* inhibitor rottlerin (4 *μ*M) for 4 h before adding ATRA (1 *μ*M) into the culture medium. Equal amount of total cell lysates were analyzed by SDS-PAGE and immunoblotted with specific antibodies against p-eIF2*α*, as described in Section 2.

To identify the signaling mechanism that regulates/activates PKR in response to ATRA or ATO we examined PKC*δ*, a serine/threonine kinase that is induced during ATRA-induced differentiation in APL cells [30, 48]. We found that ATRA and ATO induce PKC*δ* expression, which is closely correlated with the increased phosphorylation (Ser 51) of eIF2*α* in NB4 cells by Western blot analysis (Figure 5(a)). Upregulation of PKC*δ* expression was also associated with phosphorylation of PKC*δ* on threonine 505 (Thr-505) in the activation loop (Figure 5(b)). RT-PCR analysis suggested that ATRA (1 *μ*M) induces PKC*δ* expression at the transcriptional level (Figure 5(c)). ATRA also induced PKC*δ* expression also correlated with increased phosphorylation of eIF2*α* in U937 AML cells (Figure 5(d)), indicating that PKC activation is not APL cell line specific event.

To determine whether PKC*δ* plays a role in the regulation of eIF2*α* phosphorylation (Ser-51), we inhibited PKC*δ* using a PKC*δ* inhibitor rottlerin (at 4 *μ*M specifically inhibits PKC*δ*) [49, 50]. Rottlerin treatment blocked basal levels and ATRA-induced phosphorylation of eIF2*α* in NB4 cells (Figure 5(e)). Densitometric analysis revealed that inhibition of PKC*δ* led to 5-fold reduction in p-eIF2*α* levels (lower panel). Overall, these findings suggest that PKC*δ* is involved in regulation of phosphorylation of eIF2*α*.

### 3.6. PKC*δ* Regulates Phosphorylation of eIF2*α* in HSC and AML Cells

**Figure 6:**
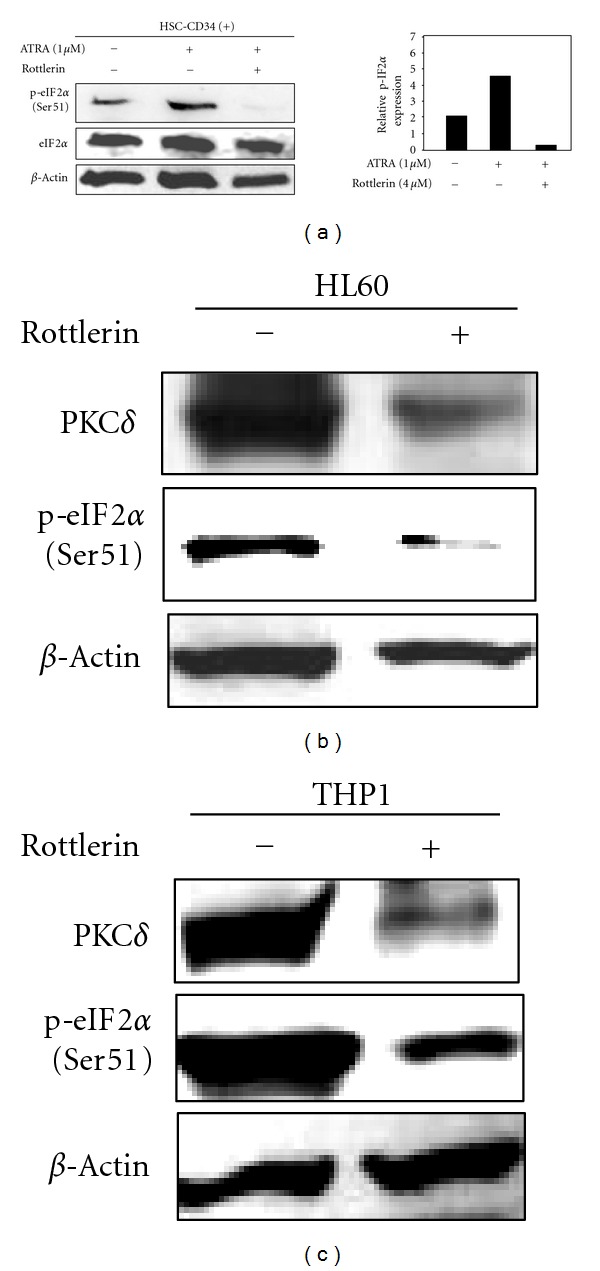
PKC*δ* mediates ATRA-induced phosphorylation (Ser51) of eIF*α* in normal CD34+HCC and AML cells. (a) All cells were treated with ATRA (1 *μ*M) in the presence or absence of rottlerin for 24 h and p-(Ser51)-eIF2*α* was examined by Western blot analysis. ATRA induced-eIF2*α* phosphorylation in human normal CD34+ bone marrow HCC cells. Densitometric analysis shows (right panel) relative expression of p-eIF2*α* protein levels after normalization to *β*-actin expression level. Inhibition of PKC*δ* by rottlerin blocked the Ser51-phosphorylation eIF2*α* in HCC cells (a), HL60 (M2-AML) (b), and THP1 (M4-AML) cells (c).

We also investigated whether PKC*δ* regulates eIF2*α* activity in normal human hematopoietic stem cells (HSC) isolated from bone marrow of a healthy donor. Western blot analysis showed that inhibition of PKC*δ* by rottlerin (4 *μ*M) markedly blocked ATRA-induced phosphorylation of eIF2*α* in HSCs, HL60 myeloblastic (AML-M2), and THP1 monocytic AML cells (AML-M5) (Figures 6(a)–6(c)). Rottlerin treatment also caused a slight inhibition of total eIF2*α*. These findings indicated that PKC*δ* plays a role in regulation of eIF2*α* not only in APL and AML as well as normal HSCs.

### 3.7. PKC*δ* Regulates Phosphorylation of eIF2*α* through PKR

**Figure 7:**
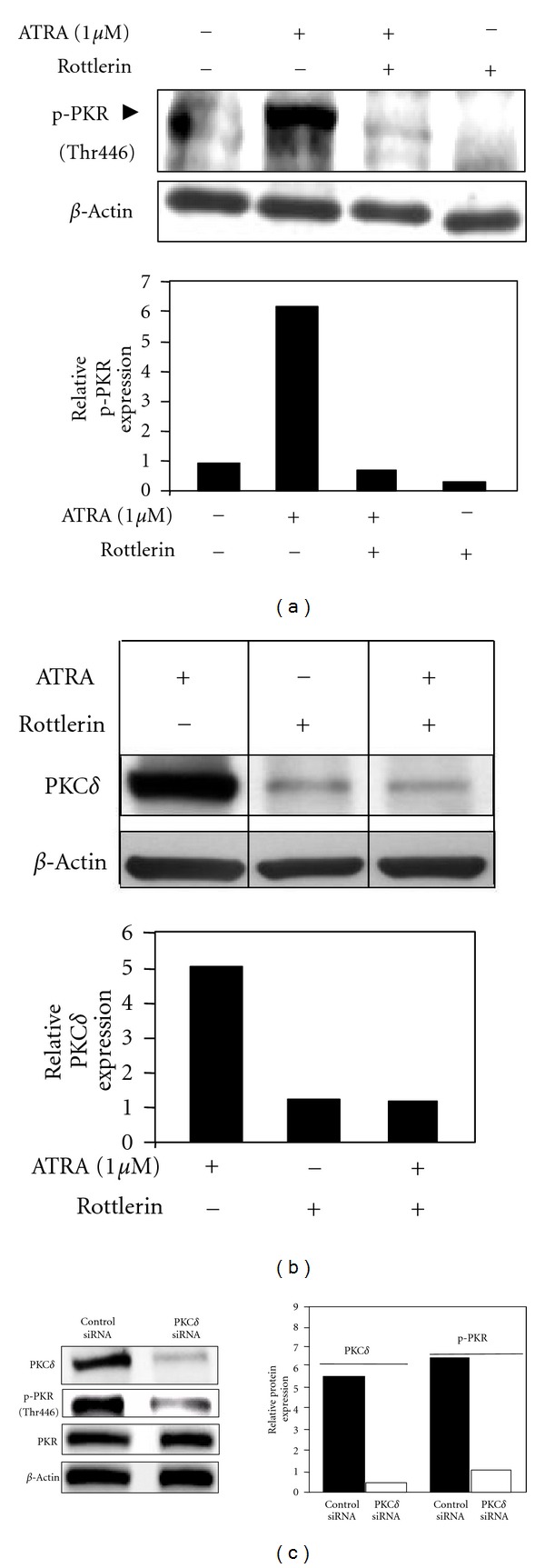
Inhibition of PKC*δ* blocks ATRA-induced PKR and Ser51-phosphorylation of eIF2*α*. (a) Inhibition of PKC*δ* blocked ATRA-induced phosphorylation (Thr446)/activation of PKR. NB4 cells were pretreated with PKC*δ* inhibitor rottlerin (4 *μ*M) for 4 h before adding ATRA (1 *μ*M). (b) Rottlerin (4 M) inhibits ATRA-induced expression of PKC*δ* in NB4 cells detected by Western blot analysis. (c) Knockdown of PKC*δ* by a specific siRNA results in reduction of phosphorylation (Thr446) of PKR in NB4 cells. Bar graph represents inhibition of relative expression of PKC*δ* (PKC*δ*/*β*-actin) and p-PKR (p-PKR/*β*-actin) based on densitometric analysis of Western blots.

PKR, a component of the antiviral defense, is of the kinases known to phosphorylate eIF2*α* at Ser51 to inhibit its activity and translation initiation and protein synthesis [25]. Because our data previously indicated that inhibition of PKR (Figure 3(f)) or PKC*δ* (Figure 5(d)) prevented basal and ATRA-induced phosphorylation of eIF2*α*, we hypothesized that PKC*δ*, a Serine/Threonine kinase, may be responsible for activation of PKR through phosphorylation on Thr446. Therefore, we next examined whether PKC*δ* regulates PKR phosphorylation. Pretreatment of NB4 cells with PKC*δ* inhibitor rottlerin significantly inhibited ATRA-induced Thr446-phosphorylation of PKR (Figure 7(a)). The bar graph next to Figure 7(a) shows densitometric analysis of Figure 7(a).  Figure 6(b) confirms that rottlerin treatment inhibited PKC*δ* in response to ATRA in NB4 cells. To provide further link between PKC*δ* and PKR we knockdown PKC*δ* by a specific siRNA and found that inhibition of PKC*δ* expression and reduced activity (phosphorylation) of PKR (Thr446) (Figure 7(c) and densitometry, right panel), indicating that PKC*δ* regulates PKR activity/phosphorylation in APL cells.

**Figure 8:**
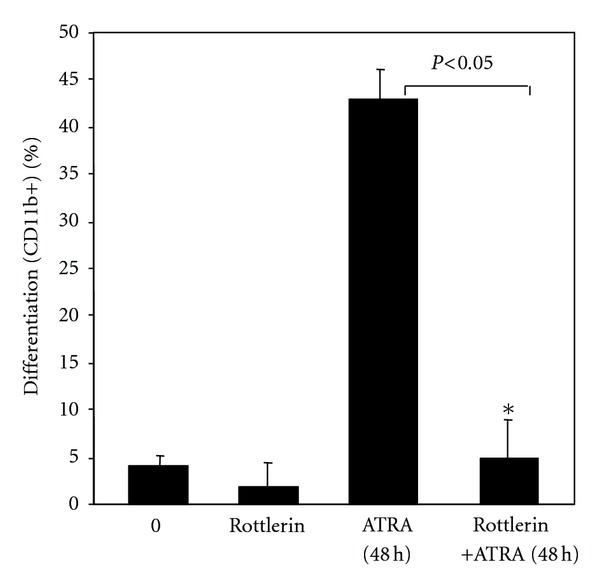
PKC*δ* plays a critical role in ATRA-induced terminal differentiation of APL cells. Inhibition of PKC*δ* blocked ATRA-induced granulocytic differentiation of NB4 cells. NB4 cells were pretreated with rottlerin (4 *μ*M) for 4 h before adding ATRA (1 *μ*M). After 48 h of ATRA treatment, cells were collected and analyzed for CD11b positivity as a measure of granulocytic differentiation.

To evaluate the role of PKC*δ* signaling in cell differentiation of APL cells we specifically inhibited PKC*δ* and assessed ATRA-induced (48 h) differentiation in NB4 cells. Inhibition of PKC*δ* by rottlerin significantly inhibited ATRA-induced granulocytic differentiation in cells (Figure 8). While ATRA (1 *μ*M for 48 h) treatment led to about 43.4% cell differentiation in NB4 cells, preincubation with rottlerin reduced ATRA-induced differentiation to 5.2% (*P* < 0.05). This finding suggested that PKC*δ* plays a key role in ATRA-induced granulocytic differentiation.

### 3.8. Inhibition of Mammalian Target of Rapamycin (mTOR) Induces p-(Ser51) eIF2*α* through PKC*δ*/PKR Axis

**Figure 9:**
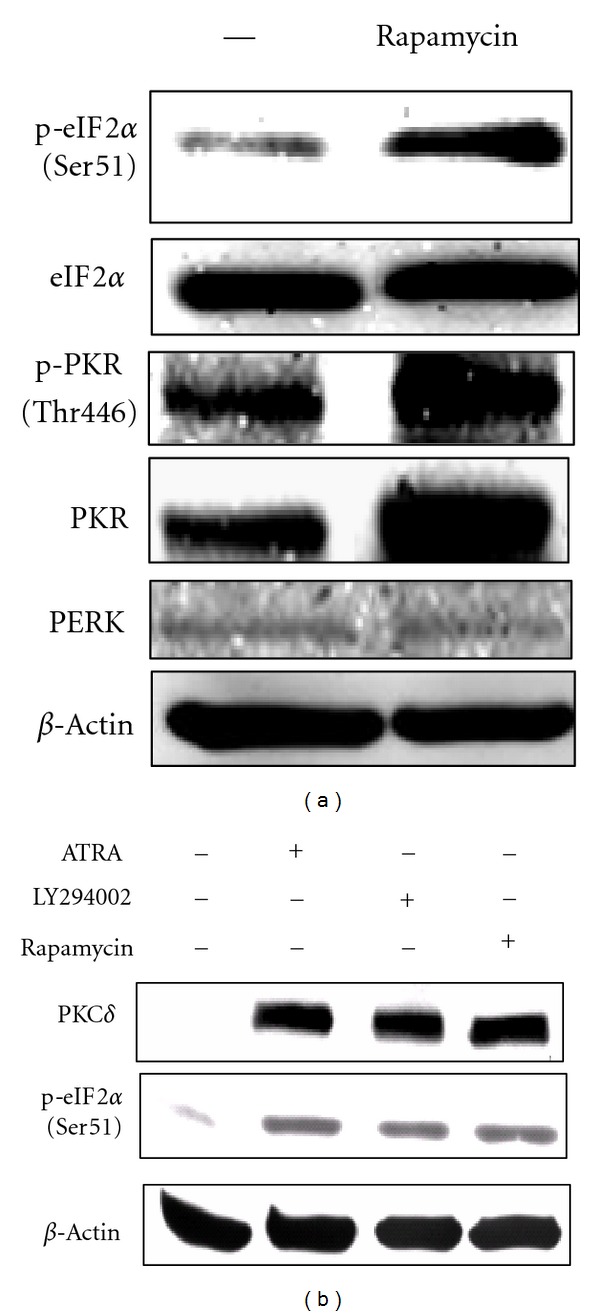
mTOR signaling is involved in regulation of (Ser51) of eIF2*α* through PKC and PKR. (a) Inhibition of PI3K/Akt/mTOR signaling pathway by rapamycin (20 nM), a specific mTOR inhibitor, led to induction of phosphorylation eIF2*α* in NB4 cells. Rapamycin treatment results in induction of p-(Ser51) eIF2*α* and PKR and p(Thr446)-PKR in NB4 cells. (b) NB4 cells were pretreated with a specific PI3K inhibitor (LY294002, 20 *μ*M) induced PKC*δ* and p-eIF2*α* levels. ATRA Equal amounts of total cell lysate were subjected to Western blot analysis to detect p-eIF2*α*, p-Akt, and PKC*δ* expression.

Inhibition of mammalian target of rapamycin (mTOR) signaling has been shown to potentiate the effects of ATRA and ATO to induce growth arrest and differentiation of APL (NB4) and AML cells in vitro and in vivo models [51, 52]. mTOR signaling is one of the major regulators of translation in cancer cells by altering 4EBP-1 and eIF4E activity [53]. We have previously shown that ATRA and ATO inhibit the activity PI3K/Akt/mTOR and p70S6 kinase [33, 34]. To investigate whether mTOR regulates eIF2*α* through PKR we inhibited the PI3K/Akt/mTOR pathway by a specific mTOR inhibitor rapamycin (20 nM). Treatment of NB4 cells with rapamycin for 24 h markedly induced p-PKR and total PKR expression that is closely associated with phosphorylation (Ser51) of eIF2*α* (Figure 9(a)). Inhibition of PI3K pathway by a specific inhibitor LY294002 (20 uM) [33] also resulted in increased p-(Ser51) eIF2*α* and PKC*δ* in NB4 cells (Figure 9(b)), indicating that the PI3K/Akt/mTOR pathway is involved in phosphorylation of eIF2*α* by PKC*δ*/PKR in APL cells.

### 3.9. eIF2*α* Phosphorylation Leads to Differential Expression of Important Cellular Proteins

**Figure 10:**
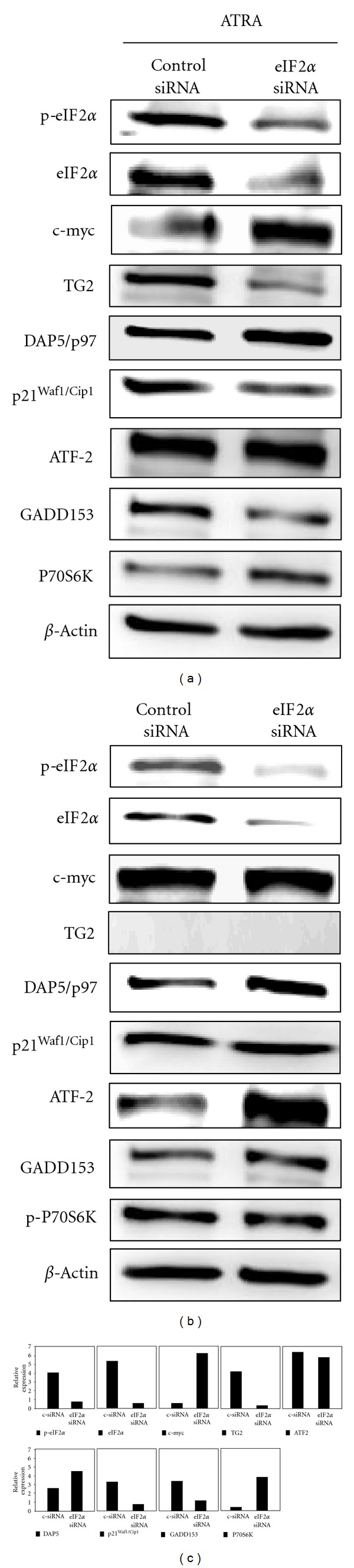
eIF2*α* is involved in ATRA-mediated expression of c-myc, DAP5, TG2, and P70S6 in NB4 cells. NB4 cells were transfected with control or eIF2*α* siRNA for 48 h in the absence (a) or in the presence of ATRA (1 *μ*M, 48 h) (b). (c) Densitometry analysis (lower panel) represents relative expression of Western blot bands after treatments with control or eIF2*α* siRNA in the presence of ATRA. Protein bands were normalized expression based actin levels.

Studies have shown that ATRA regulates important regulatory proteins such as cyclin-dependent kinase inhibitor p21^Waf1/Cip1^ [54], death-associated protein 5 (DAP5/p97) [55, 56], c-myc [23, 57], p-P70S6K [58], and TG2 [46, 47, 59]. To gain some insight about the ATRA-mediated downstream changes in response to eIF2*α* regulation we knocked down eIF2*α* by siRNA and examined if expression of these proteins is altered. Knockdown of eIF2*α* inhibited ATRA-induced upregulation of p21^Waf1/Cip1^, DAP5/p97, and TG2 (Figure 10(a)). We also observed inhibition of ATRA-induced downregulation of c-myc and p-P70S6K, a downstream mediator of PI3K/Akt/mTOR pathway (Figure 10(a)). These findings suggest that eIF2*α* in fact is involved in expression of some of the proteins involved in cell cycle arrest, proliferation, survival, differentiation, and apoptosis.

#### 3.9.1. PKC*δ* and p-eIF2*α* Protein Expressions Are Associated with Relapses in AML Patient

**Figure 11:**
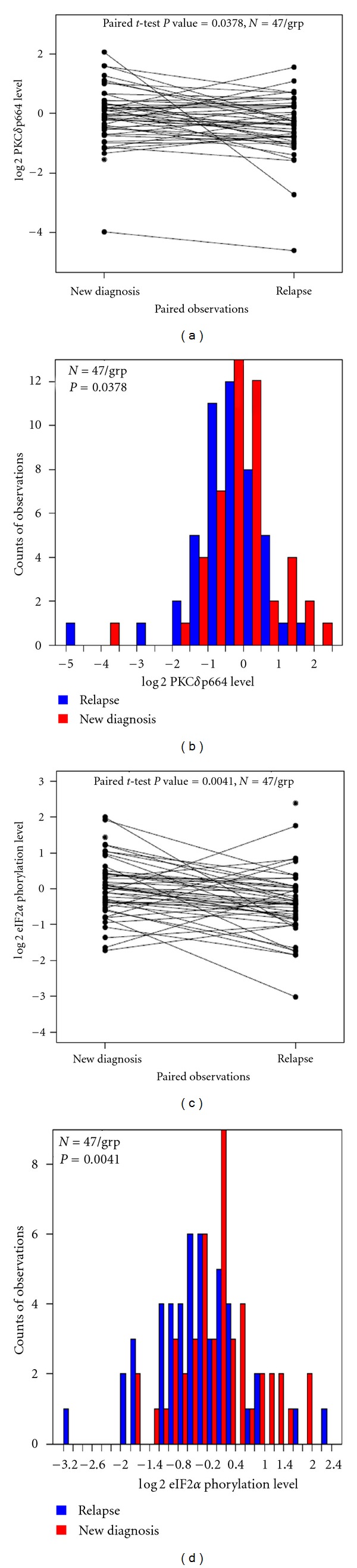
Reduced PKC*δ* and p-eIF2*α* protein expression is associated with relapses in AM patients. PKC*δ* and p-eIF2*α* protein expression was assessed by RPPA in 47 paired samples from newly diagnosed and relapsed AML patients to determine if the protein level changes when the disease status changes. The comparison of PKC*δ* and p-eIF2*α* protein expression ((a) and (c)) and distributions ((b) and (d)) of the protein levels between pairs (newly diagnosed and relapsed) were plotted. Data suggest that there is a significant association between the reduced levels of PKC*δ* (*P* = 0.0378) and p-eIF2*α* (*P* = 0.0041) and relapses in AMP patients.

PKC*δ* and p-eIF2*α* protein expression was assessed by RPPA assay in 47 paired samples from newly diagnosed and relapsed AML patients to determine if the protein level changes when the disease status changes. The comparison of PKC*δ* (Figures 11(a) and 11(c)) and p-eIF2*α* protein (Figures 11(b) and 11(d)) expression and distributions of the protein levels between pairs (newly diagnosed and relapsed) were analyzed. Data suggest that there is a significant relationship between the reduced levels of PKC*δ* (*P* = 0.0378) and p-eIF2*α* (*P* = 0.0041) and relapses in AMP patients, suggesting that higher levels of PKC*δ* and p-eIF2*α* is associated with better response.

## 4. Discussion

Overactivity of translation initiation factors, such as eIF2*α*, eIF4E, and eIF4G, results in malignant transformation, indicating that regulation of the activity of these factors is critical in controlling survival pathways and cell proliferation [6–13, 22]. Previous reports including ours suggest that growth inhibition and terminal cell differentiation are associated with suppression of translation initiation [28, 32–34, 39, 60]. However, molecular mechanisms responsible for regulation of eIF2*α* during these events have not been elucidated.

**Figure 12:**
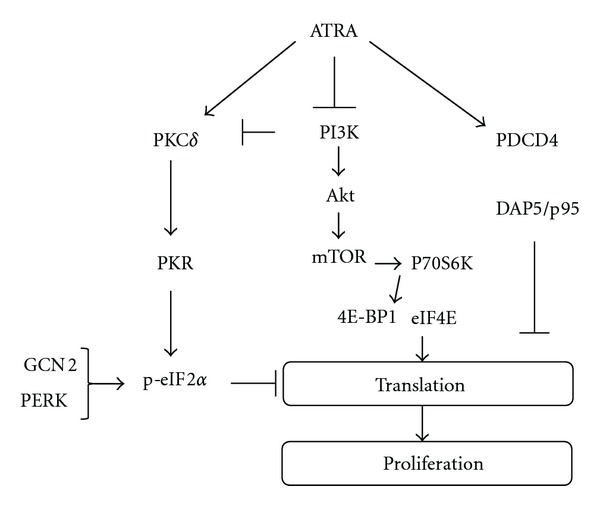
PKC*δ* is involved in regulation of translational initiation by eIF2*α* through PKR axis in AML cells. ATRA and ATO inhibit translation initiation by inducing inactivation (phosphorylation at Ser-51) of eIF2*α*. Suppression of translation initiation is also regulated by other mediators, including induction of translational suppressors (PDCD4 and DAP5) and inhibition of 4E-BP1 [32–34]. The eIF2*α* kinases other than PKR, such as GCN2 and PERK, are not involved in regulation of eIF2*α* during ATRA-induced differentiation. eIF2*α* is involved in regulation of ATRA-regulated cellular proteins in cell cycle arrest (p21^Waf1/Cip1^), proliferation (c-myc), survival (p70S6K), and apoptosis (DAP5/p97) and differentiation (TG2).

The present study provides the first evidence that PKC*δ* regulates activity of eIF2*α* through induction of PKR, an eIF2*α* kinase, but not PERK and GCN2, leading to inhibition of translation initiation during terminal differentiation of APL and AML cells (Figure 12). Moreover, our study suggested that PI3K/Akt pathway and its downstream mediator mTOR, a major hub of translational control, is involved in regulation of eIF2*α* through PKC*δ*/PKR axis. Overall our findings revealed a novel mechanistic insight on actions of ATRA and ATO in regulation of eIF2*α*, the rate limiting step of translation initiation in APL and AML cells.

PKC*δ*, a serine/threonine kinase, can function as a tumor suppressor and a proapoptotic factor and can regulate cell proliferation and cell survival functions [30]. Induction of PKC*δ* has been previously shown in APL cells [48]. PKC*δ* is critical to ATRA-induced terminal differentiation because inhibition of PKC*δ* by rottlerin resulted in almost complete blockage of ATRA-induced differentiation (Figure 8). Our data also indicated that induction of PKC*δ* signaling is critical for regulation of eIF2*α*, linking for the first time PKC*δ* with the regulation of translation initiation, which is often over activated in AML cells. Most importantly, our data suggested that PKC*δ* regulates PKR and eIF2*α* and thereby revealing a novel function of PKC*δ*.

PKR, GCN2, and PERK are eIF2*α* kinases that are known to phosphorylate and inhibit the activity of eIF2*α* [9, 25]. However, we found that ATO and ATRA treatments induce PKR but not GCN2 and PERK. The activation of unfolded protein response (UPR) is manifested by phosphorylation of protein kinase RNA-like endoplasmic reticulum kinase (PERK) and eIF2*α*. In our study we did not detect any change in the activity (or phosphorylation) of PERK on phosphorylation of eIF2*α*, suggesting that ATRA and ATO do not induce UPR response. PKR has been shown to be induced by interferon in myeloid leukemia cells [44]. Interferon is known to inhibit translation and protein synthesis during viral infections, limiting production of viral particles. It is possible that ATRA-induced interferon secretion from the cells may lead to upregulation and activation of PKR in APL by autocrine mechanisms. This hypothesis remains to be tested by future studies.

PI3K/Akt/mTOR signaling pathway is overactivated in APL and AML cells and plays an important role in proliferation, drug resistance, inhibition of apoptosis in cancer cells [50, 61]. mTOR signaling is a critical inducer of translational activity by phosphorylating 4EBP-1 and releasing eIF4E from 4EBP1 and increasing activity translation initiation complex [53]. We found that ATRA and ATO inhibits phosphorylation of 4E-BP1 in NB4 APL cells (unpublished observation). Dephosphorylated 4E-BP1 inhibits translation initiation by binding to eIF4E, which normally binds to the cap-structure of mRNA to form an initiation complex [62, 63]. Reduced phosphorylation of 4E-BP1 by ATRA facilitates its binding to eIF4E, leading to inhibition of eIF4E. We have also shown that ATRA and ATO inhibit the activity PI3K/Akt/mTOR and p70S6 kinase in APL cells [33, 34]. The current study is in agreement with our previous findings that ATRA inhibits translation initiation by multiple mechanisms, including inhibition of initiation factors and induction of PDCD4 and DAP5 (inhibitors of translation initiation), inhibition of p-4E-BP1 and EF4E (Figure 11) [32–34]. DAP5 and PDCD4, a novel tumor suppressor protein, were recently identified as inhibitors of translation initiation. PDCD4 binds to eIF4A and specifically inhibits its helicase activity [64]. DAP5 functions as a repressor of translation initiation by forming translationally inactive complexes with eIF4A and eIF3 [56]. Overall, data suggest that translation initiation and protein synthesis are suppressed by several mechanisms by ATRA and ATO in APL cells.

Several specific mRNAs have been found to be selectively regulated in response to inhibition of eIF2*α*. These selectively upregulated proteins include ATF-2, ATF-3, ATF-4, GADD34, and CHOP/GADD153 [25, 28, 65], suggesting that even during inhibition of translation some of the mRNAs are being translated. Our study suggest that eIF2*α* is involved in expression of p21^Waf1/Cip1^, DAP5, and TG2 in response to ATRA during differentiation, supporting the hypothesis that subset of mRNAs encoding critical proteins are differentially regulated by eIF2*α*. On the other hand, increased expression of eIF2*α* in response to growth induction by c-myc [23] and transformed cells [24] suggest that eIF2*α* plays a critical role in regulation of cell proliferation and is strictly regulated because of its oncogenic potential.

In conclusion, a better understanding of translation initiation and posttranscriptional mechanisms may help identify novel targets for targeted therapies. Antitumor agents such as rapamycin, a specific mTOR inhibitor, or related compounds that inhibit translation by inhibiting phosphorylation of 4E-BP1 and P70S6K have been shown to induce remissions even in AML patients with relapsed disease, suggesting that the targeted inhibition of mRNA translational pathways might offer therapeutic benefits for patients with certain malignancies.

## Supplementary Material

Supplementary Figure 1: Morphology and cellular structures of NB4 cells. Cells were analyzed by May-Grünwald-Giemsa staining (upper panels) and transmission electron microscopy (lower panels), respectively, before and after 72 h of ATRA (1 *μ*M) treatment.
Supplementary Figure 2: ATO at high concentrations induces apoptosis in APL cells. NB4 cells were treated with ATO (2 *μ*M) for 24 h and collected for Western blot analysis. ATO induced apoptosis in NB4 cells as indicated by cleavage (activation) of caspases 9, 3 and PARP cleavage.
Supplementary Figure 3: Knockdown of PERK by siRNA has no effect on phosphorylation of eIF2*α* in NB4 cells. Cells were transfected with PERK or control siRNA for 48 h. 24 h after ATRA treatment cells were collected, lysed and analyzed by Western blot.
