# Supplementary material for: PKCδ Regulates Translation Initiation through PKR and eIF2α in Response to Retinoic Acid in Acute Myeloid Leukemia Cells
Source: Leuk Res Treatment. 2012 Jul 15;2012:482905. doi: 10.1155/2012/482905 (PMC3505929; doi:10.1155/2012/482905)
Supplement: Supplementary file 1 — Supplementary Figure 1: Morphology and cellular structures of NB4 cells. Cells were analyzed by May-Grünwald-Giemsa staining (upper panels) and transmission electron microscopy (lower panels), respectively, before and after 72 h of ATRA (1 μM) treatment. Supplementary Figure 2: ATO at high concentrations induces apoptosis in APL cells. NB4 cells were treated with ATO (2 μM) for 24 h and collected for Western blot analysis. ATO induced apoptosis in NB4 cells as indicated by cleavage (activation) of caspases 9, 3 and PARP cleavage. Supplementary Figure 3: Knockdown of PERK by siRNA has no effect on phosphorylation of eIF2α in NB4 cells. Cells were transfected with PERK or control siRNA for 48 h. 24 h after ATRA treatment cells were collected, lysed and analyzed by Western blot. [file 482905.f1.pdf]

Supplementary Figures

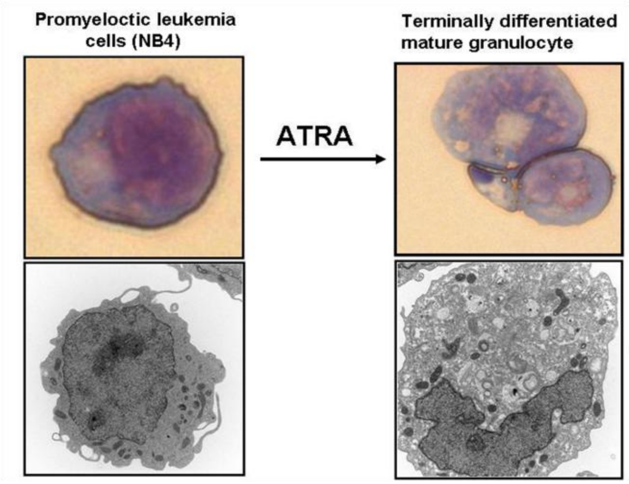

Supplementary Figure 1; ATRA induced changes in NB4 cells following May-Grünwald-Giemsa by light and electron microscopy (39).
Ozpolat *et al.,*

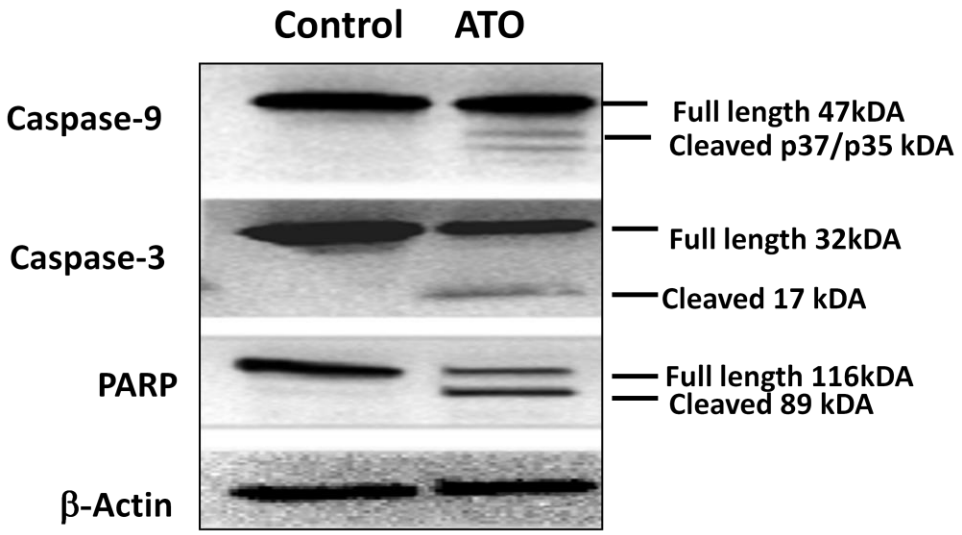

Supplementary Figure 2: ATO induces apoptosis in APL cells. Apotosis was evidenced by capspase 9, 3 and PARP cleaveage in NB4 cells by 1uM ATO. (24h)
.Ozpolat *et al*.,

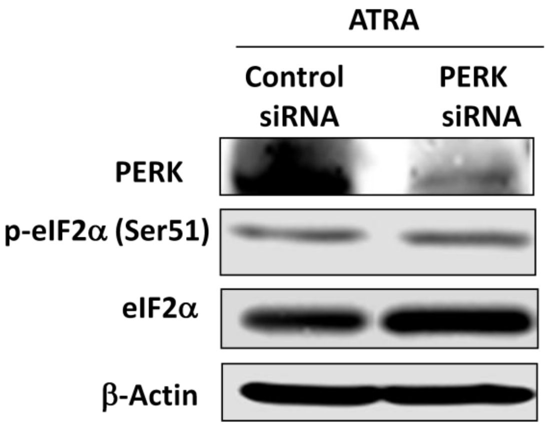

Supplementary Figure 3. Knockdown of PERK by siRNA has no effect on phosphorylation of IF2α in NB4cells. Cells were tranfected with PERK or control siRNA for 48h.
24h after ATRA treatment cells were collected, lysed and analyzed by Western blot.
